# P-461. Clinical Outcomes of Pediatric Meningitis: A Short-Term Study in a Developing Country Context

**DOI:** 10.1093/ofid/ofaf695.676

**Published:** 2026-01-11

**Authors:** Abay kassie, Alamirew Gessesse, Zelalem Anteneh, Senay Mengste

**Affiliations:** Debretabor University, Bahirdar, Amara, Ethiopia; Bahir Dar University, Bahirdar, Amara, Ethiopia; Bahir Dar University, Bahirdar, Amara, Ethiopia; Byumba Level 2 Teaching Hospital, Byumba, Nord, Rwanda

## Abstract

**Background:**

Despite improvements in medical care, the mortality and neurologic sequelae associated with acute bacterial meningitis remain high in developing countries. The objective of this study was to evaluate short-term treatment outcomes and identify factors associated with poor outcomes in this setting.

**Figure:**
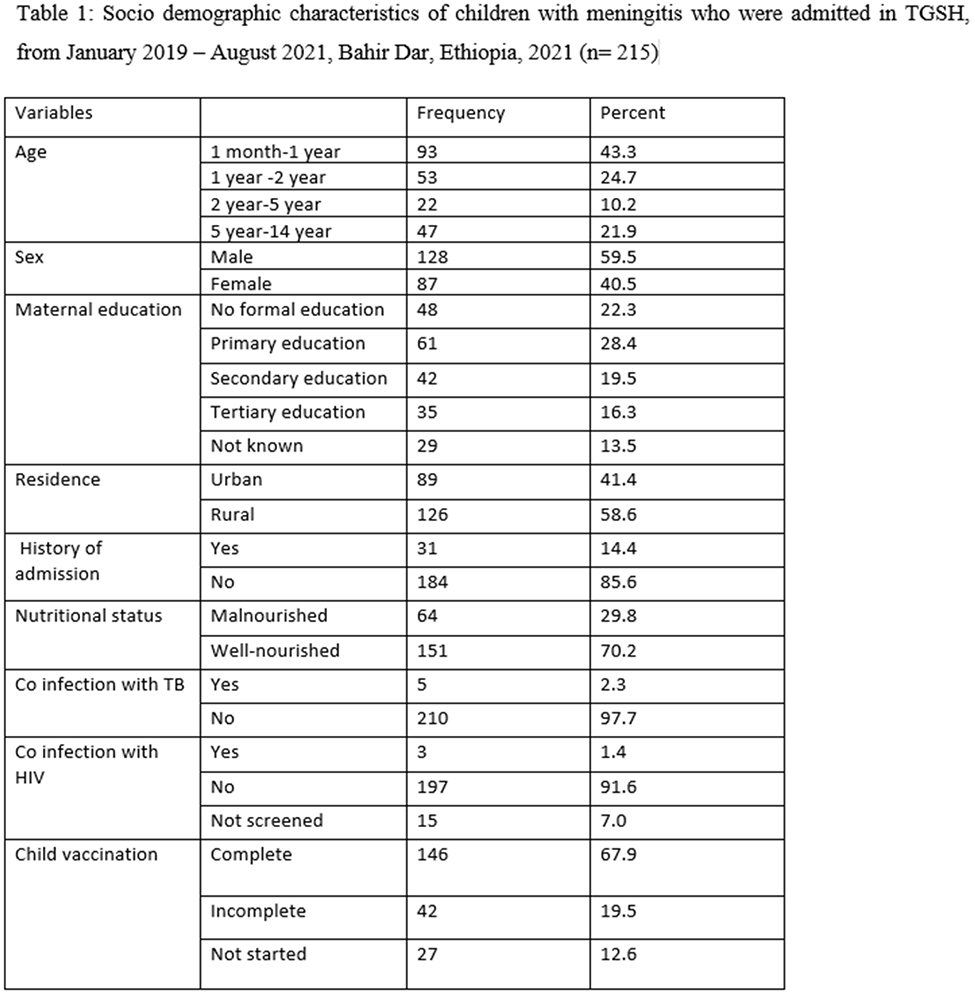

**Figure:**
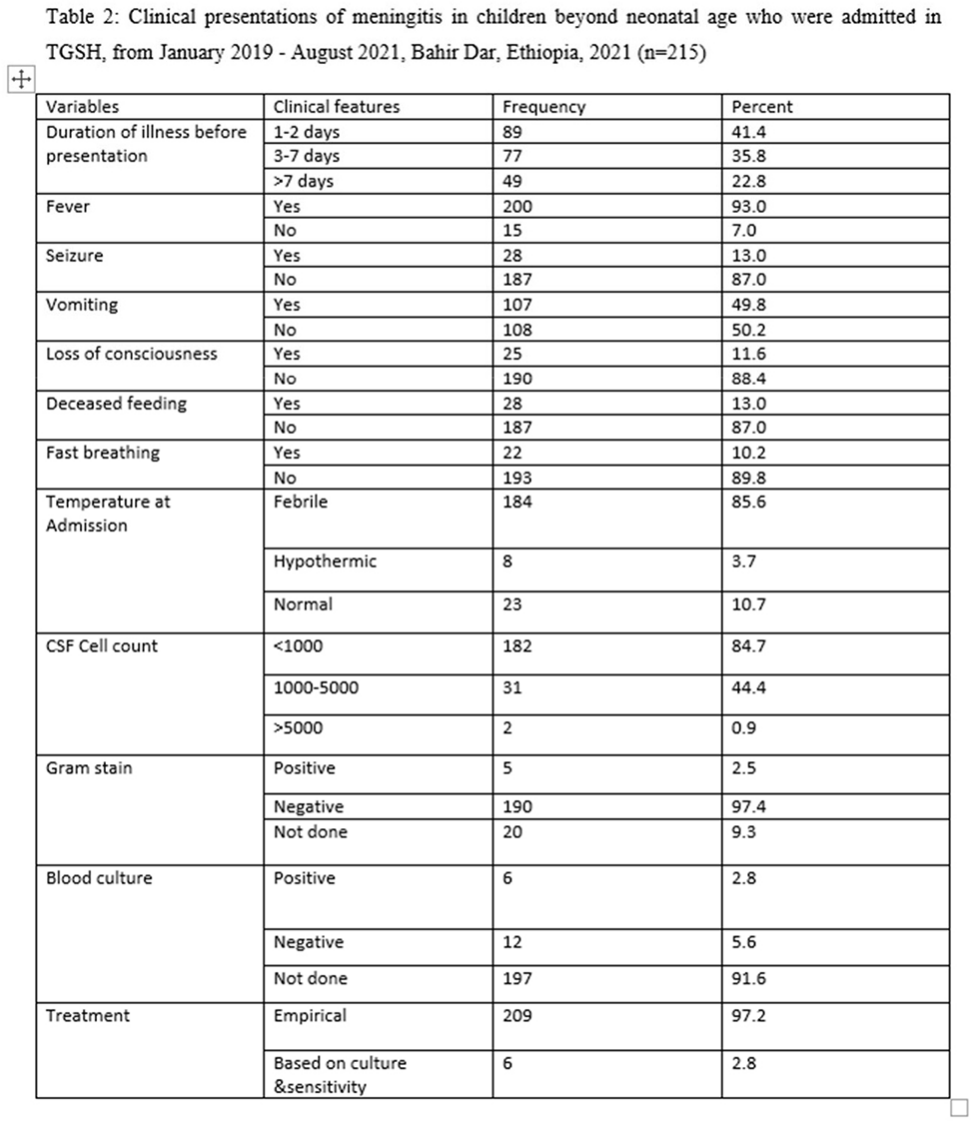

**Methods:**

A hospital-based cross-sectional study design was conducted among 215 children diagnosed with meningitis and admitted to a referral hospital. The data was collected by using structured checklists, and checked for completeness and inconsistencies before being entered into SPSS Version 25 for analysis. Associations between independent variables and dependent variables were analyzed using bivariate and multivariate analysis to identify factors that are significantly associated with the outcome variable. The findings were presented with p-values, and odds ratio with a 95% confidence level. A p-value of less than 0.05 was used to declare the significance level.

**Figure:**
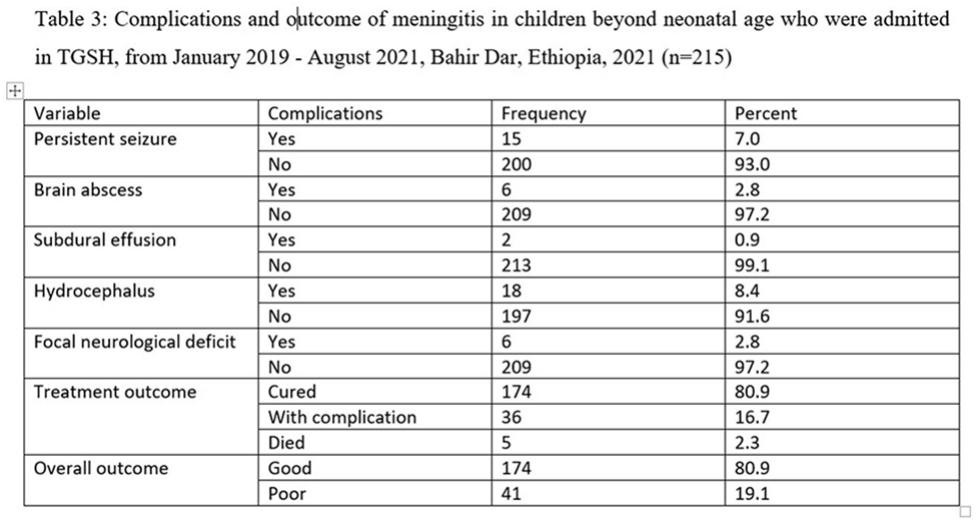

**Figure:**
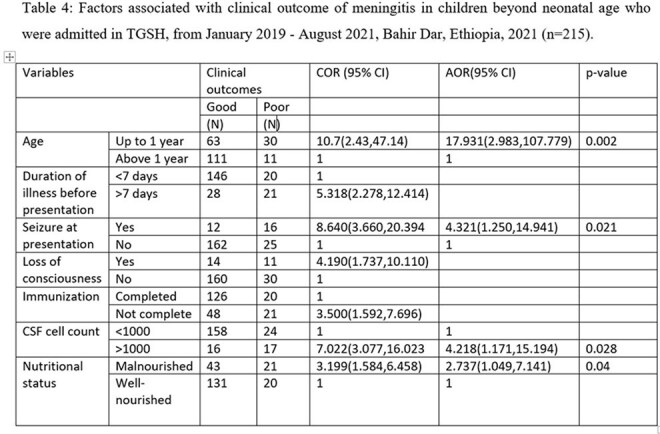

**Results:**

About 19.1% of children beyond neonatal age with meningitis developed poor outcomes, either developed complications or died. On presentation, 13% and 11.6% had seizures and impaired level of consciousness respectively. Hydrocephalus (8.4%), persistent seizure (7%), brain abscess (2.8%), focal neurologic deficit (2.8%), and subdural effusion (0.9%) were the most common complications of meningitis. Age [AOR= 17.931(2.983, 107.779)], the presence of seizure at presentation [AOR= 4.321(1.250, 14.941)], CSF cell count [AOR= 4.218(1.171, 15.194)] and poor nutritional status [AOR= 2.737(1.049, 7.141)] were the determinant factors for clinical outcome of meningitis.

**Conclusion:**

This study highlights the significantly high burden of meningitis in children both in terms of long- term complication and mortality. Notably one in five of children beyond neonatal age with meningitis developed poor outcomes, including complication or death. Key factors indicated in poor outcome of meningitis are age, the presence of seizure at presentation, CSF cell count, and poor nutritional status. This study underscores the importance of addressing risk factors like poor dietary status and diagnosing early patients to improve the outcome of meningitis and mitigate complications and mortality.

**Disclosures:**

All Authors: No reported disclosures

